# Integrating chemotherapy, radiotherapy, and O6-methylguanine-DNA methyltransferase (MGMT) status with deep-learning cellular tumor volumetry sharpens prediction of glioblastoma recurrence on postoperative MRI

**DOI:** 10.1093/noajnl/vdag178

**Published:** 2026-07-09

**Authors:** Taha Belbadaoui, Andrew Forester, Farzad Khalvati, Louis Gagnon

**Affiliations:** Department of Physics, Engineering Physics and Optics, Laval University, Quebec City, QC, Canada; Department of Physics, Engineering Physics and Optics, Laval University, Quebec City, QC, Canada; Department of Medical Imaging, Faculty of Medicine, University of Toronto, Toronto, ON, Canada; Department of Radiology and Nuclear Medicine, Laval University, Quebec City, QC, Canada

**Keywords:** diffusion MRI (restricted spectrum imaging), glioblastoma, pseudoprogression, radiomics, temozolomide

## Abstract

**Background:**

Distinguishing glioblastoma recurrence from posttreatment effects on magnetic resonance imaging (MRI) remains a major diagnostic challenge. While recent models consider O6-methylguanine-DNA methyltransferase (MGMT) status, most machine learning models addressing this problem assume uniform chemotherapy and radiotherapy exposure, overlooking real-world treatment variability. This study aimed to determine whether integrating chemotherapy, radiotherapy, and MGMT status together with automated cellular tumor volume (VolCT) improves predictive accuracy in posttreatment glioblastoma assessment.

**Methods:**

We retrospectively analyzed 135 postsurgical MRI examinations from 97 glioblastoma patients treated between January 2008 and December 2022. The dataset included 105 confirmed recurrences and 30 cases of treatment-related change. A total of 8465 radiomic features were extracted from 5 MRI sequences (T1-weighted pre/postcontrast, T2-weighted, FLAIR, and restricted spectrum imaging cellularity maps). Cellular tumor volumes were automatically segmented using nnU-Net and combined with chemotherapy radiotherapy and MGMT status. Models were trained using Extremely Randomized Trees with nested Monte Carlo cross-validation.

**Results:**

The baseline Top10 radiomic model achieved an area under the curve (AUC) of 0.765. Incorporation of nnU-Net-derived VolCT significantly improved performance to 0.809 (*P* = 2.84 × 10^−7^). Adding chemotherapy, radiotherapy, or MGMT status to the Top10+VolCT model yielded further gains, with AUCs ranging from 0.828 to 0.830 (all *P* ≤ .032). The full model combining VolCT with chemotherapy, radiotherapy, and MGMT achieved the best performance, with an AUC of 0.853 (*P* = .0006 vs Top10+VolCT).

**Conclusions:**

Incorporating chemotherapy, radiotherapy, and MGMT status improves posttreatment glioblastoma classification. Deep learning-derived cellular tumor volumetry further enhances radiomics-based performance, highlighting the value of combining clinical context with advanced computational imaging.

Key Points

- Recent chemotherapy status improves classification of recurrence vs treatment effect.
- Deep learning-based cellular tumor volumetry outperforms simple volumetric proxies.
- Integrated radiomics+volumetry+chemotherapy+radiotherapy+MGMT model achieves area under the curve of 0.853.

Importance of the Study

Accurately distinguishing glioblastoma recurrence from treatment-related effects remains a challenge on routine MRI and can delay or misdirect care. Existing radiomics and deep learning approaches typically assume uniform treatment exposure and overlook real-world heterogeneity in chemotherapy, radiotherapy, and MGMT status at the time of imaging. We demonstrate that integrating recent chemotherapy status, radiation, and MGMT status with quantitative imaging biomarkers significantly improves classification performance, and that deep learning-based cellular tumor volumetry provides gains over conventional volumetric surrogates. The combined model (radiomics + cellular tumor volume + chemotherapy + radiotherapy + MGMT) achieved the highest AUC (0.853) in Monte Carlo cross-validation across 25 splits. These findings highlight the value of coupling clinical and molecular variables with advanced computational imaging to enhance posttreatment decision-making. Because the inputs rely on standard MRI sequences and readily available clinical and molecular information, this framework is well positioned for prospective validation and translation into neuro-oncologic practice.

Glioblastoma represents the most aggressive primary brain tumor in adults, with a median survival of approximately 15 months despite maximal therapy.^1^^,^^2^ Standard treatment comprises surgical resection followed by concurrent chemoradiotherapy with temozolomide and subsequent adjuvant chemotherapy.^1^^,^^3^ However, the assessment of treatment response presents substantial challenges, as posttreatment imaging changes can closely mimic tumor recurrence on conventional magnetic resonance imaging (MRI).^4^^,^^5^ This diagnostic uncertainty complicates clinical decision-making and may delay appropriate therapeutic interventions or lead to unnecessary treatments.

The phenomenon of pseudoprogression, occurring in up to 30% of glioblastoma patients within the first 3 months after chemoradiotherapy completion, exemplifies this diagnostic challenge.^6^^,^^7^ Pseudoprogression manifests as increased contrast enhancement and perilesional edema that radiographically resembles tumor progression but represents treatment-induced changes rather than true tumor growth. This phenomenon is particularly common in patients with O6-methylguanine-DNA methyltransferase (MGMT) promoter methylation, reflecting enhanced treatment sensitivity.^8^ Similarly, radiation necrosis can develop months to years after treatment, presenting imaging characteristics virtually indistinguishable from recurrent tumor using conventional MRI sequences.^9^^,^^10^ Current clinical practice relies heavily on serial imaging, clinical assessment, and occasionally invasive procedures such as stereotactic biopsy to differentiate between these entities.^11^

Advanced MRI techniques have emerged as potential solutions to this diagnostic dilemma. Perfusion-weighted imaging, diffusion-weighted imaging, and MR spectroscopy provide physiological information beyond conventional anatomical imaging.^12^^,^^13^ Among these, restricted spectrum imaging (RSI), an advanced multishell diffusion technique, has demonstrated particular promise in quantifying tumor cellularity and distinguishing hypercellular tumor from hypocellular treatment effects.^14-16^ However, individual advanced imaging modalities have shown variable performance across different studies, suggesting that multiparametric approaches may be necessary for optimal diagnostic accuracy.^17^^,^^18^

The advent of radiomics has transformed quantitative imaging analysis by extracting high-dimensional feature sets that capture subtle tissue characteristics imperceptible to visual inspection.^19^^,^^20^ These computational approaches can quantify tumor heterogeneity, texture patterns, and morphological features that may correlate with underlying biological processes. Machine learning algorithms can subsequently identify complex patterns within these high-dimensional datasets to predict clinical outcomes.^21^ Recent studies have demonstrated the potential of radiomic analysis in various neuro-oncology applications, including survival prediction, molecular subtyping, and treatment response assessment.^22-24^

Despite these technological advances, existing radiomic and deep learning studies in glioblastoma have generally assumed relatively uniform treatment protocols.^23^^,^^25-27^ While some studies have incorporated proxy variables such as MGMT methylation status or time intervals since treatment completion,^28^ few have directly evaluated chemotherapy exposure, radiotherapy exposure, and MGMT status as explicit input variables that differ between patients. This limitation fails to capture the heterogeneity of real-world clinical practice, where patients may differ in treatment exposure at the time of imaging. The present study addresses these gaps by developing and validating a machine learning framework that integrates chemotherapy status, radiotherapy status, and MGMT status with quantitative imaging biomarkers to distinguish residual/recurrent glioblastoma from treatment-related changes.

The present study addresses these gaps by developing and validating a machine learning framework that systematically integrates clinical chemotherapy, radiotherapy, and MGMT status with quantitative imaging biomarkers for distinguishing residual/recurrent glioblastoma from treatment-related changes.

## Methods

### Study Design and Patient Cohort

The dataset used in this study is a publicly available dataset (The University of California San Diego annotated posttreatment high-grade glioma multimodal MRI dataset—UCSD-PTGBM) available on The Cancer Imaging Archive.^29^ The dataset consisted initially of 243 MRIs from 178 patients; however, only cases with available chemotherapy, radiotherapy, and MGMT status information were included in this study. The final dataset consisted of 135 timepoints from 97 patients. Patient demographics are summarized in Table 1. For patients with multiple timepoints, all timepoints were assigned to the same fold during the analysis to avoid data leakage. Classification of recurrence vs treatment-related change was based on a combination of longitudinal imaging evolution, clinical course, and multidisciplinary expert consensus. Pathologic confirmation was included when available, but most cases were adjudicated using sustained imaging progression or stability over follow-up. In our labeling framework, the presence of identifiable cellular tumor was considered recurrence, even if treatment-related changes coexisted, whereas cases classified as treatment-related change showed no evidence of viable tumor on longitudinal assessment.

**Table 1.** Patient demographics and clinical characteristics of the study cohort

Characteristic	Value
Timepoints	135
Patients	97
Age, years (mean ± SD)	56.4 ± 13.4
Male sex, *n* (%)	64 (62%)
Female sex, *n* (%)	33 (38%)
GTR	52 (53.6%)
Chemotherapy before acquisition, *n* (%)	76 (56.3%)
Radiotherapy before acquisition, *n* (%)	74 (54.8%)
MGMT positive, *n* (%)	71 (52.6%)
Recurrent disease (timepoints), *n* (%)	105 (77.8%)
Treatment-related changes only (timepoints), *n* (%)	30 (22.2%)

Abbreviation: GTR, gross total resection.

### MR Imaging Protocol

All imaging was performed on 3-Tesla scanners (GE Signa Excite HDx or GE Discovery MR750) equipped with 8-channel head coils, following the standardized brain tumor protocol at UCSD. Notably, no 1.5-Tesla studies met the RSI sequence inclusion criterion, ensuring consistency in image quality across the cohort.

The comprehensive imaging protocol incorporated multiple sequences designed to capture complementary aspects of tumor biology and treatment response.^29^ Three-dimensional T1-weighted images were acquired both before and after gadolinium administration (0.1 mmol/kg gadobenate dimeglumine or gadobutrol) to delineate enhancing tumor components. T2-FLAIR sequences provided assessment of perilesional edema and nonenhancing tumor infiltration, while conventional T2-weighted fast spin-echo images facilitated evaluation of cystic and necrotic changes.

### Radiomic Feature Extraction

Comprehensive radiomic profiling was performed using PyRadiomics version 3.0.1^30^ implemented through the SlicerRadiomics extension (RRL level 4, 85% compliance). We extracted 1693 IBSI-compliant features per imaging sequence, encompassing first-order statistics, shape descriptors, and texture features from multiple matrix families (GLCM, GLRLM, GLSZM, GLDM, NGTDM). Radiomic features were extracted separately for each MRI sequence and then concatenated into a single multisequence feature vector (8465 features) per examination across T1-weighted, postcontrast T1-weighted, T2-FLAIR, T2-weighted, and RSI sequences.

**Figure 1.**
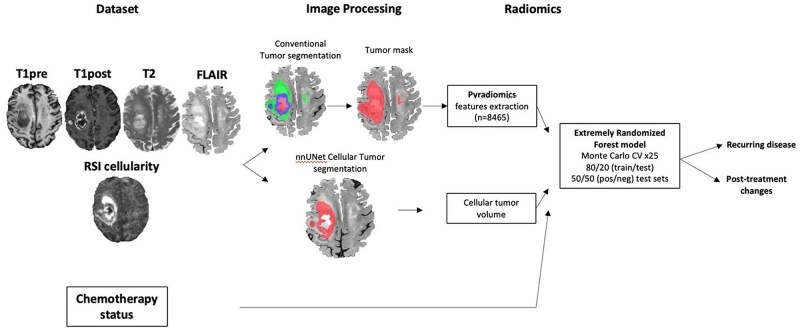
Illustration of the image processing framework and radiomic feature extraction. The conventional tumor subregion segmentation (left) includes necrotic/nonenhancing core (red), enhancing tumor (blue), and FLAIR hyperintensity (green). The tumor mask (right) represents the union of all pathological tissue components, including enhancing tumor (red) and nonenhancing/necrotic regions (green), derived from multimodal MRI sequences. The cellular tumor segmentation is obtained using an nnUNet model using multimodal MRI sequences. The radiomics features, the cellular tumor volume, and the chemotherapy status are used as input to an Extremely Randomized Forest model to distinguish recurring disease from posttreatment changes. Abbreviation: MRI, magnetic resonance imaging.

Radiomic features were computed within the whole-tumor region provided in the UCSD-PTGBM dataset, which encompasses the union of enhancing tumor, nonenhancing tumor, and necrotic components (Figure 1). PyRadiomics parameters included a fixed bin width of 25 gray levels with all feature classes and image types enabled. Accordingly, RSI radiomic features were extracted from this whole-tumor mask (enhancing + nonenhancing/necrotic) and not from a nonenhancing-only region.

In addition to traditional radiomic features, we incorporated automated cellular tumor volume (VolCT), defined as the volume of a separate nnU-Net^31^-predicted RSI tumor region of interest (ROI; ie, distinct from the whole-tumor mask) as previously described.^32^ This ROI is intended to capture cellular tumor across both enhancing and nonenhancing compartments. For comparison, we also report the standard whole-tumor radiomic volume computed from the whole-tumor mask (PyRadiomics shape feature VoxelVolume); in our feature set this whole-tumor volume is denoted as VoxelVolume_CELL (extracted during RSI feature computation), and it uses the whole-tumor mask rather than the nnU-Net ROI.

### Clinical Variables

Chemotherapy and radiotherapy were incorporated as binary treatment-exposure covariates based on their timing relative to MRI acquisition. For each scan, exposure was coded as present if the treatment had been administered before MRI acquisition and absent otherwise. Thus, scans were classified as unexposed when the treatment had not yet been given at the time of imaging, whether because it had not started yet or was delivered only later. MGMT status was included separately as a binary molecular covariate according to methylation status. This formulation allowed us to assess the incremental predictive value of these clinical and molecular variables alongside radiomic features.

### Model Development and Validation Strategy

**Figure 2.**
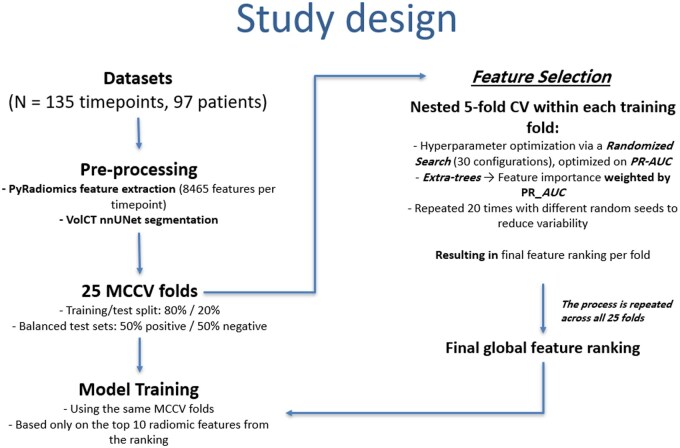
Overview of the experimental design of the study. An Extremely Randomized Trees model within a Monte Carlo cross-validation framework (25 iterations, 80/20 train-test split with balanced test sets) was used to quantify the performance of the model to detect residual/recurring tumor.

An overview of the experimental design is provided in Figure 2. We employed a Monte Carlo cross-validation framework to robustly assess model performance.^33^ Twenty-five random partitions were generated, each allocating 80% of examinations to training and 20% to testing. Each partition maintained balanced representation of outcome classes in the test set and ensured complete separation between training and testing examinations. Although each surgery-MRI pair was treated as an independent clinical timepoint, all timepoints from the same patient were assigned to the same fold during the analysis to avoid data leakage.

### Machine Learning Architecture

An Extremely Randomized Trees classifier (scikit-learn version 1.4.1) was selected as our primary modeling approach based on its robustness to high-dimensional data and ability to capture complex feature interactions.^34^ The additional randomization used by this method can help reduce overfitting compared with other tree-based approaches, while remaining well suited to nonlinear relationships among imaging and clinical variables. Compared with more data-intensive models such as convolutional neural networks, this approach is also more appropriate for limited-sample settings. Class weighting was employed to address outcome imbalance. For each Monte Carlo split, hyperparameter optimization was conducted through 5-fold inner cross-validation with random search over 30 parameter combinations (Table 2).

**Table 2.** Hyperparameter search space for Extremely Randomized Trees optimization

Parameter	Search values
Number of estimators	150, 250, 350
Maximum features	√*n*, log2(*n*), all features
Maximum depth	None, 10, 20
Minimum samples for split	2, 5, 10

Average precision (area under the precision-recall curve [PR-AUC]) served as the optimization metric given its sensitivity to performance on the minority class. The optimal configuration from inner cross-validation was retrained on the complete training partition before evaluation on the held-out test set.

### Feature Selection and Model Variants

A 3-stage feature ranking pipeline was implemented within each training partition to identify stable and predictive radiomic signatures, with all procedures confined to training data to prevent information leakage. Feature ranking was performed on the pooled multisequence (T1-pre, T1-post, T2, FLAIR, RSI) radiomic matrix (all sequences grouped). First, performance-weighted importance scores were computed from Extremely Randomized Trees models tuned by randomized search with stratified inner cross-validation exclusively within the training partition:


( (1)) $$w=1+0.5(\text{PR}-{\text{AUC}}_{\text{CV}}-0.5)$$

where PR-AUC_CV_ represents the average precision achieved during inner cross-validation, ensuring that test set information remained completely isolated from the feature-selection process.

After computing performance-weighted importance scores, the ranking procedure was repeated across multiple random initializations (*n*_stability_) to mitigate dependence on any single seed. Within each repetition, stability was quantified from top-selection *k* frequency and mean rank when selected, while consistency was estimated by permuting each top candidate feature on an internal stratified validation split (derived exclusively from training data) and measuring the associated PR-AUC decrease. Importance, stability, and consistency were then max-normalized and combined using a weighted average to produce unified per-fold rankings.

Consensus rankings across all folds guided the construction of predictor variants (Table 3), enabling systematic evaluation of feature combinations and clinical variable integration. This approach ensured that feature-selection decisions were based solely on training data patterns, preserving the integrity of the independent test set evaluation.

**Table 3.** Model variants evaluated in the study, incorporating different combinations of radiomic features, volumetric measures, and clinical variables

Model variant	Feature composition
Top10Pyrad^a^	Global top 10 radiomic features
Top10Pyrad + VolCT	Top10Pyrad + nnU-Net cellular tumor volume
Top10Pyrad + VolCT + Chemo	Top10Pyrad + nnU-Net cellular tumor volume + chemotherapy status
Top10Pyrad + VolCT + Radiation	Top10Pyrad + nnU-Net cellular tumor volume + radiotherapy status
Top10Pyrad + VolCT + MGMT	Top10Pyrad + nnU-Net cellular tumor volume + MGMT
Top10Pyrad + VolCT + MGMT + Chemo + Radiation	Top10Pyrad + nnU-Net cellular tumor volume + radiotherapy status + chemotherapy status + MGMT
Top10Pyrad + VoxelVolume_CELL	Top10Pyrad + PyRadiomics voxel volume from RSI cellularity maps
Top10Pyrad + VoxelVolume_CELL + Chemo + MGMT + Radiation	Top10Pyrad + PyRadiomics voxel volume from RSI cellularity maps + chemotherapy status + MGMT + radiotherapy status

a Top10Pyrad features (all from T1-weighted postcontrast): (1) wavelet-LLL_glcm_SumEntropy_T1post, (2) gradient_glcm_SumEntropy_T1post, (3) wavelet-LHL_glcm_DifferenceAverage_T1post, (4) original_glcm_SumEntropy_T1post, (5) gradient_gldm_DependenceEntropy_T1post, (6) wavelet-LLL_glcm_JointEntropy_T1post, (7) gradient_glcm_JointEntropy_T1post, (8) wavelet-LHL_glcm_DifferenceEntropy_T1post, (9) original_glcm_JointEntropy_T1post, (10) wavelet-LHL_glrlm_RunEntropy_T1post.

### Model Variants, Performance Assessment, and Statistical Analysis

To evaluate the incremental value of volumetric and clinical variables, we systematically tested model variants incorporating different combinations of Top10 radiomic features, volumetric measures, chemotherapy status, radiation status, and MGMT status. Specifically, we compared models combining the Top10 radiomic baseline with either automated VolCT or whole-tumor voxel volume, with and without the addition of the clinical covariates. This design enabled quantification of each component’s contribution to overall model performance.

Model performance was evaluated using area under the receiver operating characteristic curve (ROC-AUC) as the primary metric, quantifying the ability to discriminate between residual/recurrent tumor and treatment-related changes. Area under the precision-recall curve provided complementary assessment accounting for class imbalance. Performance metrics were computed on each held-out test partition, with results reported as mean ± standard error of the mean (SEM) across 25 Monte Carlo iterations. Comparative performance between model variants was assessed using 1-tailed paired *t*-tests on ROC-AUC values across Monte Carlo splits. The directional hypothesis posited that enriched models would outperform baseline configurations. Statistical significance was defined at α = 0.05, with exact *P-*value reported in the “Results” section.

## Results

### Model Performance Overview

Using the updated cohort including chemotherapy, radiation, and MGMT covariates, the baseline Top10 radiomics model achieved a mean ROC-AUC of 0.765 and a mean PR-AUC of 0.777 across 25 Monte Carlo cross-validation iterations. Adding the VoLCT derived from the nnU-Net segmentation model (VolCT) improved performance to an ROC-AUC of 0.809 and a PR-AUC of 0.825, representing a significant improvement over the Top10 model for both metrics (paired *t*-test: ROC-AUC, *P* = 3 × 10^−7^; PR-AUC, *P* = 3 × 10^−7^).

**Figure 3.**
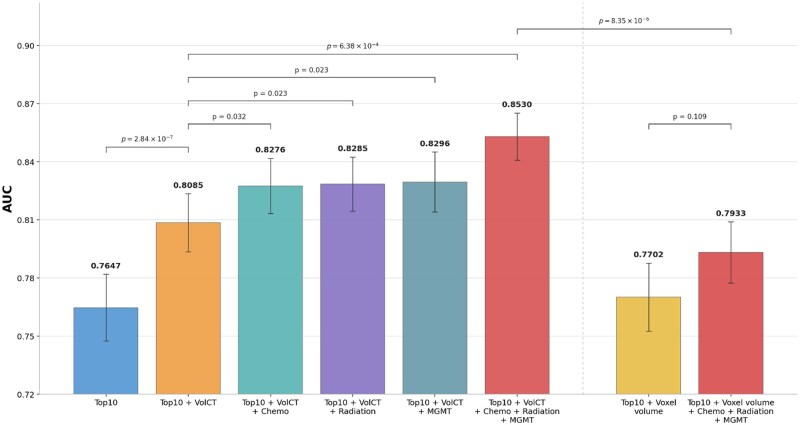
ROC-AUC performance comparison across the selected 8 model variants. Bars represent mean ROC-AUC across 25 Monte Carlo cross-validation iterations, error bars indicate SEM, and *P-*value correspond to paired *t*-tests across iterations.

**Figure 4.**
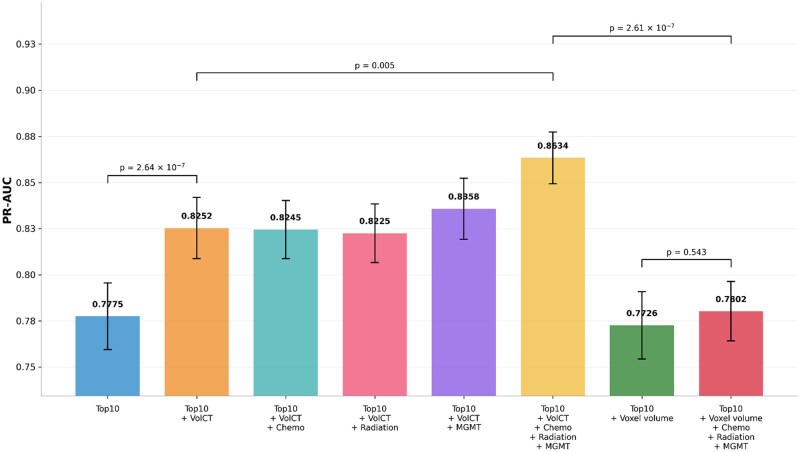
PR-AUC performance comparison across the selected 8 model variants. Bars represent mean PR-AUC across 25 Monte Carlo cross-validation iterations, error bars indicate SEM, and *P-*value correspond to paired *t*-tests across iterations.

The highest overall performance was achieved by the most comprehensive VolCT-based model, which combined Top10 radiomic features with VolCT, chemotherapy, radiation, and MGMT status. This model reached a mean ROC-AUC of 0.853 and a mean PR-AUC of 0.863, significantly outperforming both the Top10 baseline (ROC-AUC, *P* = 3 × 10^−6^; PR-AUC, *P* = 1 × 10^−6^) and the Top10+VolCT model (ROC-AUC, *P* = 6 × 10^−4^; PR-AUC, *P* = .005). An overview of ROC-AUC performance is shown in Figure 3, and the corresponding PR-AUC results are shown in Figure 4.

### Integration of VolCT and Clinical Covariates

Within the VolCT-based branch, adding individual clinical covariates yielded modest but consistent gains in ROC-AUC. Top10+VolCT+Chemo, Top10+VolCT+Radiation, and Top10+VolCT+MGMT achieved mean ROC-AUC values of 0.828, 0.829, and 0.830, respectively, each significantly higher than Top10+VolCT alone (all *P *< .05). In contrast, the PR-AUC results were more heterogeneous. Top10+VolCT+Chemo and Top10+VolCT+Radiation achieved PR-AUC values of 0.824 and 0.823, respectively, which were similar to Top10+VolCT (0.825), whereas Top10+VolCT+MGMT showed the strongest individual PR-AUC increase (0.836).

Taken together, these findings suggest that the addition of single clinical covariates provides incremental benefit, but that the clearest improvement is obtained when chemotherapy, radiation, and MGMT are incorporated jointly. Indeed, the combined Top10+VolCT+Chemo+Radiation+MGMT model outperformed all other VolCT-based variants and provided the best overall discrimination in both ROC-AUC and PR-AUC.

### Comparison Between VolCT and Voxel Volume

To assess whether a simpler volumetric surrogate could replace the segmentation-derived VolCT was substituted with the PyRadiomics shape feature voxel volume. The Top10+VoxelVolume model achieved a mean ROC-AUC of 0.770 and a mean PR-AUC of 0.773, which did not significantly differ from the Top10 baseline and remained significantly inferior to Top10+VolCT (ROC-AUC, *P *= 6 × 10^−5^; PR-AUC, *P *= 3 × 10^−7^).

Adding chemotherapy, radiation, and MGMT to the voxel-volume model increased performance to an ROC-AUC of 0.793 and a PR-AUC of 0.780. However, this improvement was not significant relative to Top10+VoxelVolume for either ROC-AUC (*P *= .109) or PR-AUC (*P *= .543). Moreover, the corresponding VolCT-based model remained clearly superior, with ROC-AUC values of 0.853 vs 0.793 (*P *= 8 × 10^−6^) and PR-AUC values of 0.863 vs 0.780 (*P *= 3 × 10^−7^).

Overall, these results indicate that segmentation-derived VolCT provides information that is not captured by the simpler voxel-volume surrogate, and that this advantage persists even after the inclusion of chemotherapy, radiation, and MGMT covariates.

## Discussion

This study systematically evaluates the integration of treatment-related and molecular clinical context into machine learning models for distinguishing residual/recurrent glioblastoma from treatment-related changes. We demonstrate that incorporating chemotherapy, radiation, and MGMT status provides biologically relevant information that improves diagnostic performance beyond a radiomics-only baseline. Our findings show that a parsimonious model based on 10 selected radiomic features already achieves clinically meaningful discrimination, but that performance is further improved by the addition of segmentation-derived VolCT and, most clearly, by the joint incorporation of chemotherapy, radiation, and MGMT. Across both ROC-AUC and PR-AUC, the most comprehensive VolCT-based model achieved the highest overall performance. These results support a multimodal approach in which quantitative imaging biomarkers are interpreted in conjunction with treatment and molecular context in the challenging posttreatment setting.

### Synergistic Value of Integrating Chemotherapy Status With Radiomic Features

The robust performance of our baseline radiomic model underscores the inherent value of quantitative imaging features in characterizing post-treatment glioblastoma. The dominance of T1-weighted postcontrast features among the highest-ranked variables aligns with their established role in capturing enhancement patterns associated with blood-brain barrier disruption. At the same time, the additional gains observed after incorporation of volumetric and clinical variables indicate that radiomic information alone does not fully capture the biological complexity of posttreatment lesions.

Several previous studies have attempted to incorporate clinical data alongside imaging features, although the specific variables and integration strategies have varied. Jang et al^26^ developed a hybrid CNN-LSTM model combining MRI features with clinical inputs including age, sex, radiotherapy dose and fractions, interval from chemoradiotherapy, and molecular markers such as MGMT promoter methylation and IDH mutation status, achieving an AUC of 0.83. Similarly, Gomaa et al^35^ developed a self-supervised Vision Transformer approach integrating postcontrast T1-weighted and FLAIR MRI with clinical parameters and radiotherapy treatment-planning information through guided cross-modal attention, achieving an AUC of 75.3% on an external test set. Sun et al^23^ evaluated clinical factors including age, Karnofsky score, and extent of resection, but found none significantly predictive in their cohort, leading to a purely radiomics-based model.

In our updated analysis, the contribution of clinical variables was more nuanced than a uniform gain across all model configurations. Within the VolCT-based branch, the addition of individual covariates yielded modest improvements in ROC-AUC, whereas PR-AUC changes were more heterogeneous. The clearest improvement was observed when chemotherapy, radiation, and MGMT were incorporated jointly, suggesting that treatment history and molecular context provide complementary rather than redundant information. This finding is clinically relevant because posttreatment glioblastoma is not interpreted in a biological vacuum: lesion appearance is influenced not only by intrinsic tumor features, but also by prior and ongoing therapy, as well as by treatment responsiveness reflected in markers such as MGMT.

Previous approaches have often captured treatment effects indirectly through proxy variables such as MGMT methylation status, a predictor of temozolomide responsiveness, or through temporal variables such as the interval between chemoradiotherapy and lesion appearance.^26^ While these markers are informative, our results suggest that modeling chemotherapy exposure, radiation exposure, and MGMT together offers a broader and more clinically faithful representation of treatment context than relying on any single surrogate alone. This may be particularly important in real-world cohorts, where treatment intensity, interruptions, and response patterns are more heterogeneous than in tightly controlled trial populations.

The superior performance of the combined Top10+VolCT+Chemo+Radiation+MGMT model supports a multimodal strategy for posttreatment glioblastoma assessment. In this framework, radiomic features provide structural and textural information, volumetric measurements summarize disease burden in a biologically targeted way, and clinical variables contextualize imaging findings within the therapeutic trajectory of the patient. Taken together, these complementary sources of information appear to offer a more accurate representation of the processes underlying posttreatment imaging changes than any single modality alone.

### Volumetric Measurements and Segmentation Strategies

Our results distinguish the value of a cellular tumor specific volume from that of a simpler volumetric surrogate. The nnU-Net-derived VolCT improved discrimination relative to the baseline Top10 radiomics model and consistently outperformed the simpler voxel-volume measure. Importantly, this advantage persisted even after incorporation of chemotherapy, radiation, and MGMT. In other words, the performance gap between VolCT and voxel volume was not eliminated by the addition of clinical variables, indicating that the 2 measures are not interchangeable.

These findings suggest that the biologically targeted measurement of VolCT captures information beyond lesion size alone. Conceptually, VolCT is designed to emphasize enhancing and nonenhancing cellular tumor components while excluding regions such as necrosis or nonspecific FLAIR hyperintensity, which may dilute the specificity of broader volumetric estimates. This likely explains why VolCT retained added value across both ROC-AUC and PR-AUC, whereas the simpler voxel-volume surrogate showed more limited gains and did not achieve comparable performance even in the clinically enriched model.

From a translational perspective, 2 practical implications emerge. First, when cellular tumor segmentation is available, incorporation of VolCT is preferable because it provides the most consistent improvement in diagnostic performance. Second, when such segmentation is unavailable, the voxel-volume surrogate may still serve as a pragmatic alternative, but it should not be considered equivalent to VolCT. In our cohort, the addition of chemotherapy, radiation, and MGMT to the voxel-volume model improved performance numerically, but this did not fully close the gap with the corresponding VolCT-based model. These observations support continued efforts to develop reliable segmentation tools that extract biologically meaningful tumor subvolumes rather than relying only on simpler geometric descriptors.

### Methodological Considerations

Our Monte Carlo cross-validation framework with balanced test sets addresses several methodological challenges inherent to radiomics research. By ensuring equal representation of outcomes in each test set, we mitigate optimistic bias that can arise from class imbalance. This contrasts with previous studies that have employed simpler validation strategies. For example, Moassefi et al^25^ used a standard 5-fold cross-validation scheme without additional class-balancing techniques, whereas Sun et al^23^ relied on internal 5-fold cross-validation with SMOTE oversampling but did not include an independent test cohort. By evaluating our models across 25 stratified patient-level splits, we obtained more stable performance estimates than would be expected from a single train-test partition or a limited number of validation folds. The fact that the main ordering of model performance was preserved across both ROC-AUC and PR-AUC further supports the robustness of the central conclusions.

The feature-selection strategy used in this study also contributes to methodological rigor in a high-dimensional radiomics setting. Compared with approaches such as LASSO-based feature selection^24^ or end-to-end deep learning architectures that bypass explicit feature selection,^26^ our pipeline emphasizes stability and predictive value while preserving model interpretability. By conducting all feature selection strictly within the training partitions, we maintained separation between model development and model evaluation, thereby limiting information leakage and reducing the risk of overly optimistic performance estimates.

Our choice of Extremely Randomized Trees as the primary classifier also offers practical advantages. Although deep learning approaches such as the 3D DenseNet-121 used by Moassefi et al^25^ or the Vision Transformer architecture used by Gomaa et al^35^ can capture complex patterns, they generally require larger datasets and greater computational resources. The multicenter study by Elshafeey et al^36^ also reported strong performance using traditional machine learning. In contrast, our ensemble tree-based approach offers a balance between predictive performance, interpretability, and suitability for moderately sized clinical datasets.

Another methodological strength of the present work is the explicit evaluation of clinically accessible treatment and molecular variables within the same radiomics-volumetry framework. Prior studies have incorporated some of these factors, including MGMT and radiotherapy-related information,^26^^,^^35^ but they were not always assessed in a way that clarified their incremental contribution relative to imaging features alone. Our results suggest that treatment and molecular heterogeneity contain predictive information that is not fully captured by radiomics or volumetry alone, and that future work in this area should move beyond assumptions of uniform treatment exposure when developing clinically applicable models.

### Limitations and Future Directions

Several limitations warrant consideration when interpreting these findings. First, the retrospective single-institution design limits generalizability to other clinical settings with different imaging protocols and patient populations. External validation across multiple institutions will therefore be essential before broader clinical deployment can be considered. Second, our dependence on RSI-derived inputs may limit immediate applicability, as this advanced diffusion technique is not universally available. Future work should assess whether similar performance can be achieved using more widely available diffusion sequences or in model variants that do not depend on RSI-based information.

Third, although all train-test splits were performed at the patient level to prevent information leakage, multiple postoperative MRI timepoints from the same patient were treated as separate observations, which may still lead to some overrepresentation of certain patients and may therefore affect generalizability. In addition, our reference standard was not exclusively pathology-based, but instead relied on longitudinal imaging evolution, clinical course, and multidisciplinary expert consensus. Although this reflects common real-world practice, it introduces some uncertainty compared with histopathologic confirmation. At the same time, restricting analysis to biopsy-proven cases alone could bias the dataset toward lesions that are already highly suspicious or clinically selected for intervention.

Despite these limitations, the performance achieved by the integrated models suggests that this approach is promising for prospective clinical evaluation. A decision-support tool based on such models could assist radiologists and neuro-oncology teams in interpreting complex posttreatment imaging, potentially reducing interobserver variability and improving diagnostic confidence. The ability to combine quantitative imaging features with clinically accessible treatment and molecular variables also facilitates potential integration into real-world radiology workflows.^37^

Future research should focus on external validation, prospective evaluation, and assessment of clinical utility in decision-making settings. Additional work should also explore more granular treatment variables, particularly radiotherapy timing and dosimetry, chemotherapy timing and intensity, and their interactions with molecular markers such as MGMT. The present findings suggest that joint modeling of these factors is more informative than considering them in isolation. Finally, improved interpretability methods, including feature attribution and case-level explanations, may help increase clinical acceptance by clarifying how individual predictions are generated.^38^

## Conclusions

This study demonstrates that integrating treatment-related and molecular clinical context, including chemotherapy, radiation, and MGMT status, enhances machine learning-based assessment of posttreatment glioblastoma beyond a radiomics-only baseline. Although the baseline model achieved clinically meaningful performance, the highest discrimination was obtained by combining selected radiomic features with segmentation-derived VolCT and jointly modeled clinical covariates. In addition, the superior performance of the VolCT-based models relative to the simpler voxel-volume surrogate suggests that biologically targeted volumetric characterization provides information not captured by conventional shape-based measures alone. Taken together, these findings support a multimodal framework for posttreatment glioblastoma evaluation that combines advanced imaging biomarkers with clinically accessible treatment and molecular variables.

## Data Availability

All imaging data analyzed in this study are publicly available from The Cancer Imaging Archive as the UCSD-PTGBM dataset (https://doi.org/10.7937/fwv2-dt74).^29^
